# Antiapoptotic and chemotaxis-stimulating effects of poly (d, l-lactide-co-glycolide)-chitosan and whey proteins against aflatoxicosis-induced splenic and thymic atrophy

**DOI:** 10.1007/s11033-023-08902-7

**Published:** 2023-10-15

**Authors:** Ghada M. Ezzat, Abdel-Raheim M. A. Meki, Fatma Y. Meligy, Hend Omar, Ahmed Y. Nassar

**Affiliations:** 1 Department of Medical Biochemistry and Molecular Biology, Faculty of Medicine, Assiut University, Assiut, 71515 Egypt; 2 Biochemistry Department, Faculty of Pharmacy, Sphinx University, New Assiut, Egypt; 3 Department of Restorative Dentistry and Basic Medical Sciences, Faculty of Dentistry, University of Petra, Amman, 11196 Jordan; 4 Department of Histology and Cell Biology, Faculty of Medicine, Assiut University, Assiut, Egypt; 5 Animal Health Research Institute, Assiut, Egypt

**Keywords:** Camel whey protein microparticles, Chemokines, NF-κB, Lymphocytes, Cleaved caspase-3, Aflatoxicosis

## Abstract

**Background:**

Aflatoxin B (AFB) induces toxicological effects on the liver and immune organs. The whey proteins can modulate the immune response during aflatoxicosis. Our work evaluates the novel polylactic acid-glycolic acid-chitosan-encapsulated bovine and camel whey proteins against AFB-induced thymic and splenic atrophy in rats.

**Methods and results:**

Seventy adult male Wister albino rats were divided into a control healthy group (G1) and six AFB1-intoxicated groups (G2–G7). One of the following supplements: distilled water, camel whey proteins (CWP), bovine whey proteins, poly (d, l-lactide-co-glycolide) (PLGA)- chitosan-loaded with camel whey protein microparticles (CMP), PLGA-chitosan loaded with bovine whey protein microparticles (BMP), and PLGA-chitosan nanoparticles were administered as prophylactic supplements to AFB1-intoxicated groups. The AFB-treated group showed significantly higher hepatic levels of oxidative stress and lower levels of antioxidants. In the aflatoxicated group, atrophy of the splenic lymphatic nodules and disfigurement in the organisation with an apparent decrease in the thickness of the cortex in the thymus were observed, as well as a decrease in splenic and thymic CD4+T and CD8+T lymphocytes. Moreover, CXCL12 levels were downregulated, whereas tumour necrosis factor-alpha, nuclear factor kappa B, and cleaved caspase-3 levels were upregulated. CWP, BMP, and CMP supplements markedly decreased oxidative stress, inflammation, and apoptosis, as well as significantly raised CXCL12, CD4+T, and CD8+T cells.

**Conclusions:**

The CWP, BMP, and CMP supplements rescue the liver and immune tissues from the toxic effects of AFB through their antioxidant, antiapoptotic, anti-inflammatory, and chemotaxis-enhancing roles.

**Supplementary Information:**

The online version contains supplementary material available at 10.1007/s11033-023-08902-7.

## Introduction

Aflatoxin (AF), a mycotoxin, is a severe global health risk to both humans and animals [1]. People in Africa are exposed to high doses of aflatoxin-contaminated food, particularly in Nigeria and Egypt [2]. Aflatoxicosis has been reported to suppress or modulate both adaptive and cell-mediated immunity [3].

Aflatoxicosis can disturb the structure of the immunological tissues, such as the spleen and thymus. Also, AFB results in the shrinkage of lymphoid organs by reducing lymphocyte numbers. Suppression of the antioxidant enzymes and an increase in MDA cause inflammation, cell necrosis, and subsequently apoptosis [4]. A recent study revealed that aflatoxicosis induces immunotoxicity in the jejunum of mice via modulation of differentially expressed mRNAs, long non-coding RNA, and chemokines [5].

NF-κB is an essential transcription factor, not only activated by oxidative stress but also involved in inflammation, immunity, cancer, and cell proliferation [6, 7]. NF-κB controls immune molecules and dendritic cell functions [8]. The chemokine stromal cell-derived factor-1 (SDF-1) or CXC ligand 12 (CXCL12) is a crucial myelopoietic regulator [9]. CXCL12 controls the recruitment and migration of lymphocytes [10]. Thymocyte maturation and migration from the thymus are both influenced by CXCL12 [11]. CXCL12 can also prevent oxidative stress by limiting the generation of mitochondrial free radicals [12].

Different therapeutic drugs have been identified for aflatoxicosis treatment in humans and animals. Compared to other whey proteins and bovine whey proteins (BWP), camel whey proteins (CWP) have higher biological activity [13]. Camel whey proteins influence several immune cell processes, such as increasing lymphocyte chemotaxis and cytokine release, which gives them immunologic, bactericidal, and viricidal capabilities [14] and repairing immune organ damage via encouraging B and T cell chemotaxis to lymphoid tissues [15]. Also, CWP improves oxidative stress and protects against inflammation [16].

Some bovine whey proteins (BWP) are immune-active and resistant to digestion, including α-lactalbumin, which regulates macrophages and B and T cell function, as well as β-lactoglobulin, which stimulates the proliferation of spleen cells [17]. BWP can also prime human neutrophil chemotaxis, degranulation, and superoxide generation, which can strengthen the body’s defence [17].

Lactoferrin (Lf), a glycosylated globular protein, is the most significant protein in camel milk. By blocking nuclear factor-kappa B (NF-κB) signalling, it has an anti-inflammatory effect on immune cell differentiation and migration [18]. APCs like dendritic cell, macrophage, and B cell, which transmit the processed antigen to CD4+T cells via the major histocompatibility complex II (MHC II), are also produced under the control of lactoferrin [18].

Different-sized natural nanoparticles and nanofibers serve as drug delivery methods [19]. Microencapsulation of nanoparticles is a promising method for shielding hydrophobic bioactive molecules from potentially hazardous circumstances [20]. The ability of a biodegradable polymer nanoparticle to load proteins and transport them through the intestinal barrier has been successfully tested using chitosan-coated polylactic acid-glycolic acid (PLGA) nanoparticles [21].

Based on previous reports, this study was thus undertaken to investigate the whey proteins and the whey proteins-loaded nanoparticles immunoenhancing effect against aflatoxicosis.

## Experimental procedures

### Extraction, purification, and identification of AFB

A fungal isolate, *Aspergillus flavus*, was obtained from the Centre of Fungi Research and Botany Department, Faculty of Science, Assiut University. A medium containing potatoes and dextrose was used for cultivation at 28 °C for about two weeks. Isolation and purification of the crude toxin extract were performed by thin-layer chromatography (TLC) with chloroform-methanol (97:3). Long-wave UV light (365 nm) was used for visualisation of the developed plates, where AFB was the major component in the mixture and had a bright blue fluorescence [22].

### Isolation of CWP and BWP

Raw milk was collected from the same locality and transformed into skim milk by centrifugation for half an hour at 5000 g. Acidification to pH 4.3 using 1 N HCL, then centrifugation at 10,000 g for 10 min, were applied for casein precipitation. Finally, saturation with 80% ammonium sulphate precipitated the whey proteins (WPs) [23], which were kept at − 20 °C in freeze-dried form.

### Identification of CWP and BWP by SDS-PAGE

The CWP and BWP were identified with SDS-PAGE. Electrophoresis was carried out at 110 volts, 120 ambers, and for 2 h. Coomassie brilliant blue G-250 solution was used for staining of the gels, and then 5% acetic acid discolouring was done. The protein bands in whey proteins were correlated with results obtained by previous studies [24], [25].

### Preparation of camel and bovine whey proteins PLGA-CS microparticles

PLGA-CS microparticles from poly (d, l-lactide-co-glycolide) (PLGA), polyvinyl alcohol, and chitosan (CS) were prepared by the method of the El-Kharrag et al. study [26] with some modifications. Then, the CWP and BWP-loaded PLGA-CS MPs were prepared by adding these proteins to the mixture before emulsification. The characterization of the CMP and BMP was performed by the entrapment efficiency measurement, which utilised a microplate reader (BMG Labtech, Germany), while the particle morphology of the CMP and BMP was examined by transmission electron microscopy (TEM, Philips CM-10, FEI Inc., Hillsboro, OR, USA). Particle size and zeta potential of the MPs were determined by photon correlation spectroscopy (PCS) using a Zeta Sizer (Nano ZS, Malvern Instruments, UK).

### Animals and experimental design

The Central Animal House of the Faculty of Veterinary Medicine at Assiut University provided a total of 70 adult male Wister albino rats. Their weights were about 120–150 g. The Assiut University Faculty of Medicine Ethical Committee approved all animal procedures that adhered to the regulations for the care and use of experimental animals (approval number: 17300358) for the purpose of controlling and supervising animal experiments. The authors complied with ARRIVE guidelines.

Before the experiment began, all rats were given two weeks to acclimatise in metal cages in a room with good ventilation. The animals were kept in a conventional laboratory setting with a 12-hour light/dark cycle, a temperature of 23 °C, a relative humidity of 60–70%, and a supply of water available at all times. The duration of the experiment was 4 weeks, and the treatments were given day after day by oral gavage. AFB intoxication was 3 times per week (day after day), and dietary supplements were also 3 times per week on the following day of AFB intoxication. Rats were assigned into seven experimental groups, each including 10 rats: Group I was a healthy control group that received DSMO. Groups II–VII were intoxicated with AFB (500 µg/kg) according to a previous study [27] and received one of the following supplements: distilled water (AFB-treated group), 200 mg /kg of CWP (AFB+CWP-treated group), 200 mg/kg of CMP (AFB+CM-treated group), 200 mg/kg of BWP (AFB+BW-treated group), 200 mg/kg of BMP (AFB+BMP-treated group) and 200 mg/kg of PLG-CS Ns (AFB+NPs-treated group). All groups received the same amount of supplement per dosage, which was limited to 250 µl.

### Tissue sampling

One day following the final treatment, cervical dislocation was used to kill rats from the various groups. The thymus and spleen were quickly removed, cleaned with a saline solution, and divided into three equal pieces. One piece was fixed in 10% formalin for histopathologic and immunohistochemical analyses; the second piece was embedded in liquid nitrogen and kept frozen at − 80 °C until RNA extraction; and the third piece was preserved in RIPA lysis buffer for western blot analysis. Prior to the analyses of oxidants and antioxidants, the liver samples were stored at − 20 °C.

### Oxidants and antioxidants assay in the liver

The total protein in liver homogenate was determined using a bio-diagnostic kit (Spinreact S.A.U./ Spinreact, S.A., Ctra. Santa Coloma, 7 E-17,176 Sant Esteve de Bas (Girona) ESPANA, Catalogue No. 265). Utilising the Greiss reagent and a spectrophotometer set to 540 nm, nitric oxide (NO) was measured [28]. When malondialdehyde (MDA) reacts with thiobarbituric acid at 95 °C for 30 min, the resulting colour is detected at 532 nm [29]. Using Ellman’s reagent, the GSH levels were determined Dithiobisnitrobenzoic acid (DTNB), which produces a yellow colour on reaction with glutathione peroxidase (GPx), was used to measure GPx [30]. GST was measured spectrophotometrically at 340 nm [31]. Microgramms were used to express the results per gramme of dry tissue.

### RNA isolation and quantitative real-time PCR

Using GENEzol reagent (Geneaid, Taiwan, Catalogue No. GZR100), total RNA was isolated from thymus and spleen tissues. Using the BIO-RAD T100 thermal cycler and Topscript TM cDNA synthesis kit (Enzynomics), Catalogue No. (EZ005M), as directed by the manufacturer, cDNA was produced from RNA with 1.9–2 purity. 2RT-qPCR was performed using the Applied Biosystems 7500 Fast Real-Time PCR System, USA (Catalogue No. 4351104) to determine the relative mRNA expression of the investigated genes. Thermo Fisher Scientific (Thermo, US) provided the primers. The supplementary table contains a list of the primer sequences that were employed [27]. The Primer Quest tool from Integrated DNA Technologies® (Illinois, USA) was used to create the primers. About 1 µl of the primer was taken (from each forward and reverse) in a reaction volume of 20 µl. The programming cycle used for QRT-PCR was initial denaturation at 95 °C for 10 min, 40 cycles of 95 °C for 30 s, 58 °C for 60 s, and an extension of 72 °C for 45 s. GAPDH was used as an internal reference gene. Relative mRNA expression of the target genes was calculated using 2^−△△Ct^ [32].The formulas used to be as follows: △Ct = Ct target gene-Ct internal reference gene. △△Ct =△Ct of the treated group −△Ct control group.

### Immunoblotting detection of cleaved caspase-3

For immune-blotting of cleaved caspase-3, the spleen and thymus tissues were used to make whole cell and mitochondrial extracts [20]. Tissues from the spleen and thymus (~ 3 mm^3^) were lysed in an ice-cold RIPA buffer [50 mM Tris-Cl (pH 7.6), 5 mM EDTA, 150 mM NaCl, 0.5% NP-40, and 0.5% Triton-X-100] containing 1 µg/ml leupeptin and aprotinin and 0.5 mM phenylmethylsulfonyl fluoride (PMSF). Lysates were centrifuged at 2500 rpm for 10 min at 4 °C. At 4 °C, the lysates were centrifuged for 10 min at 2500 rpm. The Bradford assay was used to quantify protein concentrations. SDS-PAGE was used to separate 40 µ g of protein into aliquots, which were subsequently transferred to nitrocellulose membranes using 10% gels. Primary antibodies (anti-cleaved caspase-3 and anti-actin 1:1000) were used to probe the membranes after they had been blocked with 5% skim milk and incubated at 4 °C overnight. Following that, membranes were incubated for 1 h at room temperature with an HRP-conjugated secondary antibody (1:10,000). The ECL substrate was used to carry out the detection. Imag J software was used to measure band densities and normalise them to β-actin.

### Immunohistochemistry

Thymus and spleen samples were fixed in 10% buffered formalin. The immunoperoxidase method was used for immunohistochemical staining. Four-millimetere-thick paraffin sections were dewaxed in graded ethanol and given two PBS rinses. A methanol/peroxide solution (0.03%) was used to suppress endogenous peroxidase activity for 30 min at 37 °C. Incubating the slices in a solution containing 1.5% normal goat serum in PBS prevented nonspecific reactions. Following that, goat polyclonal anti-CD4 and anti-CD8 antibodies were diluted to 1:200 and 1:100, respectively, and incubated with the sections for a whole night at 4 °C. Following a third PBS washing, the samples were exposed to the appropriate antibodies for a one-hour incubation period at room temperature.

### Histopathological examinations

For histological analysis, the thymus and spleen tissues were obtained. According to the Drury and Wallington procedure, tissues were stained with hematoxylin and eosin (H&E) for examination by light microscopy after being fixed in 10% neutral buffered formalin solution, embedded in paraffin wax, and cut into 5 μm-thick slices [33].

### Statistical analysis

GraphPad Prism 8 was used to analyse the data. Data is displayed as mean S.E.M. The one-way analysis of variance (ANOVA) test and the LSD test for post hoc analysis were used to determine the statistical significance between groups. Statistics were considered to be significant for differences with p-values under 0.05.

## Results

### Description of whey proteins

After the purification of milk types to obtain whey proteins, we performed Native-PAGE electrophoresis for both camel and bovine whey proteins to detect the protein bands and identify the dominant protein band in each type of whey protein. The molecular weights of the four camel whey protein bands in the two camel milk samples were identified as lactoferrin (75 kDa); camel serum albumin (66.0 kDa); lactoperoxidase (43 kDa); and lactalbumin (14.0 kDa). The most prominent electrophoretic zone in camel whey was lactoferrin content which constituted 33% of camel whey proteins, so CWP considers it a valuable source of this protein (supplementary Fig. 1A). The bovine milk samples exhibited the same four different electrophoretic patterns: lactoferrin, which has a molecular weight of 76.9 kDa; lactalbumin, which has a molecular weight of 14 kDa; and bovine serum albumin (BSA), which has a molecular weight of 66 kDa. The most noticeable electrophoretic zone in samples of bovine whey was lactalbumin, which makes up 32.7% of the proteins (supplementary Fig. 1B).

### Camel and bovine microparticles (CMP, BMP) characteristics

To show the shape of the synthetic camel and bovine proteins in microparticles (MPs), we employed transmission electron microscopy (TEM). The TEM image of CMPs is shown in supplementary Fig. 2A. Spherical MPs with a PLGA and CS nanocapsule are depicted in the figure. The TEM picture of BMPs is displayed in supplementary Fig. 2D. The image shows spherical MPs with a microcapsule of PLGA and CS. Dynamic light scattering (DLS) was used to characterize camel and bovine proteins loaded with PLGA-CS MPs. Their Z-average sizes were 1046 nm and 1040 nm, according to the DLS analysis, as shown in supplemental Fig. 2B, E. The polydispersity index (PDI) for the MPs was 0.1 and 0.1, respectively, indicating remarkable stability. 

Zeta potential measurements showed that camel proteins loaded with PLGA-CS had a consistent pattern of positive charge (ZP = + 8.13 mV), whereas bovine proteins loaded with PLGA-CS had a consistent pattern of negative charge (ZP = − 16.7 mV) (supplementary Fig. 2C, F). The entrapment efficiency percentage (EE%) of camel and bovine whey proteins in CMP and BMP was calculated using a dialysis tubing method. According to the findings, the EE% for CMP and BMP was 85.5% and 87.4%, respectively. The synthesised CMP and BMP’s zeta potential and PDI showed that the two micro-particles were stable.

### The impact of AFB and preventative whey proteins on the levels of oxidative stress in hepatic tissue

**Fig. 1:**
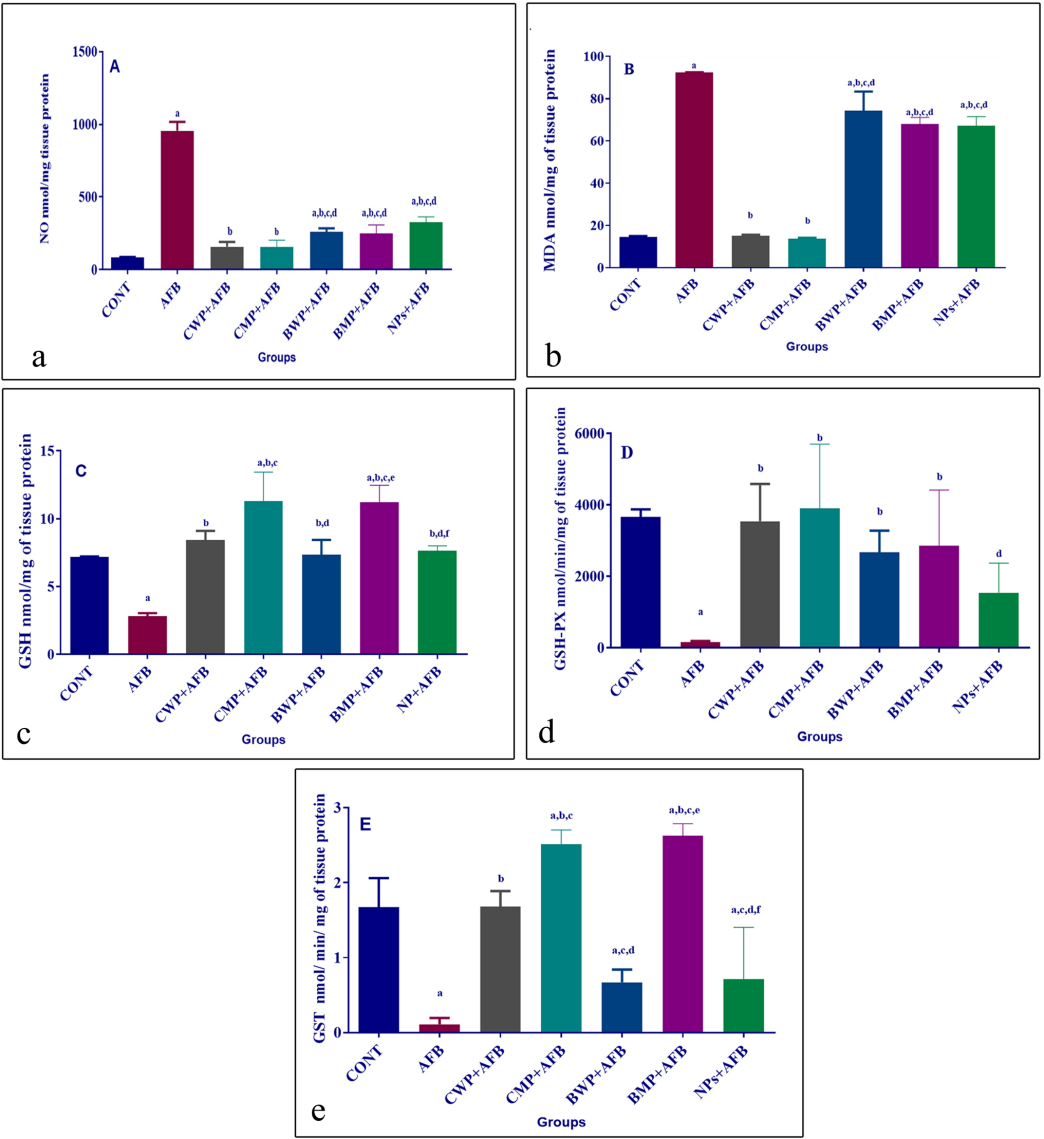
Effects of AFB intoxication and different whey proteins prophylactic treatments on NO (**A**), MDA (**B**), GSH (**C**), GSH-PX (**D**), and GST (**E**) levels in the liver. Data are presented as means ± SEM. (n = 10). a, b, c, d, e, f significance difference from CONT group, AFB, CWP+AFB, CMP+AFB, BWP+AFB, BMP+AFB, and NPs+AFB-treated groups. The one-way analysis of variance with LSD post hoc test was used for analysis of the significance between groups. *AFB* aflatoxin B, *BMP* bovine whey protein PLGA-chitosan microparticles, *CMP* camel whey protein PLGA chitosan microparticles, *CONT* control group, *GSH* reduced glutathione, *GST* glutathione-S-transferase, *GSH-PX* glutathione peroxidase, *MDA* malondialdehyde, *NO* nitric oxide, *NPs* PLGA-chitosan nanoparticles

The hepatic levels of NO and MAD were statistically increased in the AFB group in comparison to the healthy group (p < 0.001). The prophylactic doses of CWP, CMP, BWP, BMP, and NPs reduced NO and MAD levels. The CWP and CMP supplements succeeded equally in returning NO and MAD levels to those of the control group; their reductive activity was higher than that of other supplements. Notably, BWP antioxidant ability was similar to BMP supplements (Fig. 1A, B).

Aflatoxicosis resulted in a decline in the levels of the hepatic antioxidant molecules GSH, GST, and GSH-XP, while the four supplements significantly increased the antioxidant levels, but the NPs-treated groups increased only the GSH level in comparison to the aflatoxicosed group. Regarding GSH-XP levels, there were no discernible variations between the whey protein-treated groups. The AFB+CMP and AFB+BMP-treated groups had higher GSH and GST levels than those of the AFB+CWP, AFB+BWP, and AFB+NPs-treated groups (Fig. 1B–D, respectively).

### The effect of AFB and whey proteins on CXCL12, NF-κβ, and inflammatory cytokines relative mRNA expression in the thymus

**Fig. 2:**
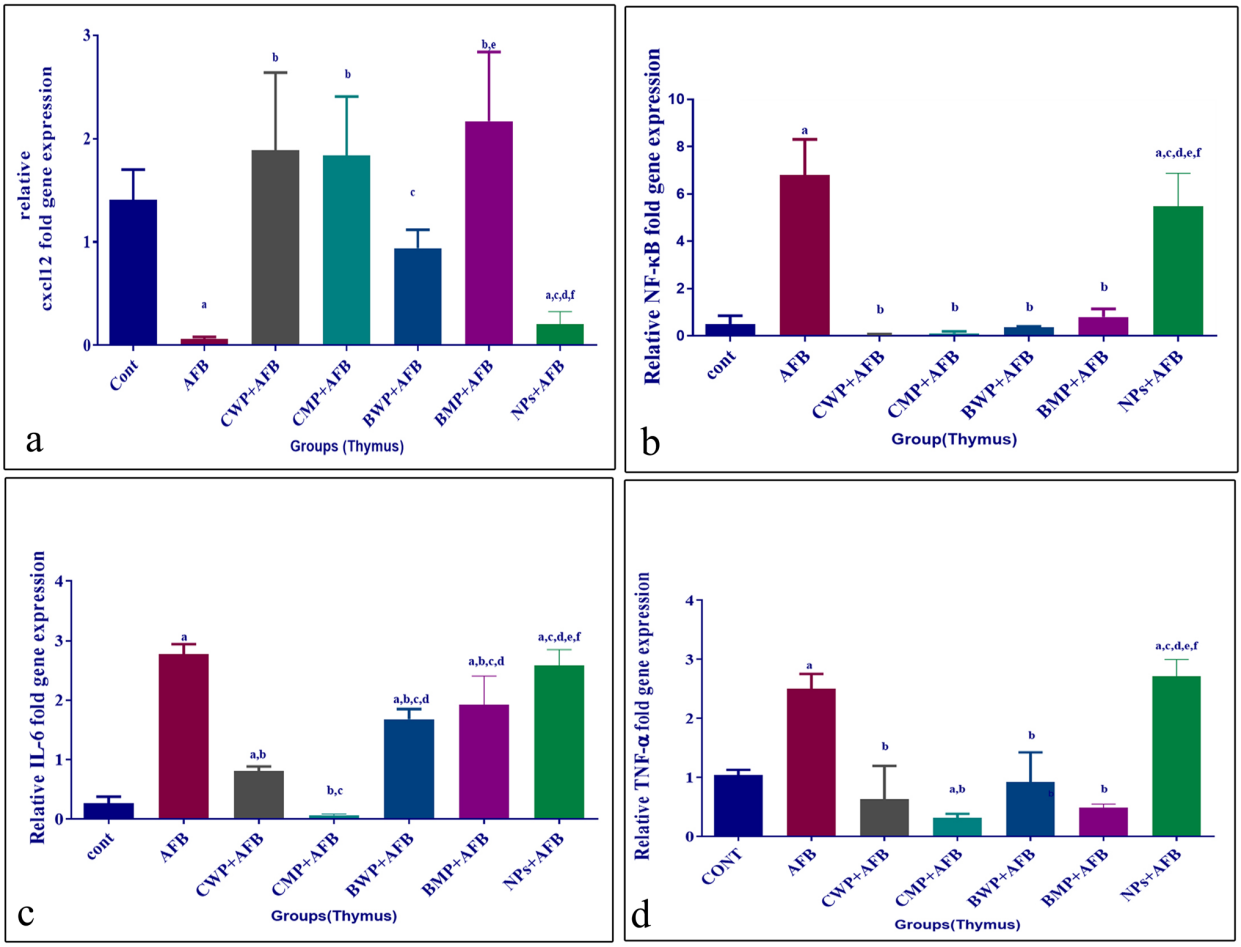
Effects of AFB intoxication and different whey proteins prophylactic treatments on the relative expression of CXCL12 (**A**), NF-κβ (**B**), IL-6 (**C**), and TNF-α (**D**) in the thymus. Data are presented as means ± SEM. (n = 10). a, b, c, d, e, f significance difference from CONT group, AFB, CWP+AFB, CMP+AFB, BWP+AFB, BMP+AFB, and NPs+AFB treated groups. The one-way analysis of variance with LSD post hoc test was used for analysis of the significance between groups. GAPDH is a control gene. *AFB* aflatoxin B, *BMP* bovine whey protein PLGA-chitosan microparticles, *CMP* camel whey protein PLGA chitosan microparticles, *CONT* control group, *CXCL12* Chemokine-x-C 12, *IL-6* Interleukin-6, *NF-κβ* Nuclear factor-Kappa β, *NPs* PLGA-chitosan nanoparticles, *TNF-α* Tumor necrosis factor-α

CXCL12 expression in the thymus was downregulated by AFB intoxication and was significantly increased by CWP, CMP, and BMP treatments, but not significantly by BWP (Fig. 2A). mRNA thymic levels of TNF-α and NF-kβ were increased by AFB and decreased by both whey proteins and whey protein microparticles treatments to a similar extent (Fig. 2B, D), whereas the inflammatory cytokine IL-6 was also significantly increased by AFB intoxication and was declined by the five treatments; however, the CWP and CMP supplements showed significantly lower levels than BWP and BMP supplements, and the CMP treatment effect on IL-6 levels was significantly higher than the CWP treatment (Fig. 2C).

### The effect of AFB and whey proteins on CXCL12, NF-κβ, and inflammatory cytokines relative mRNA expression in the spleen

**Fig. 3:**
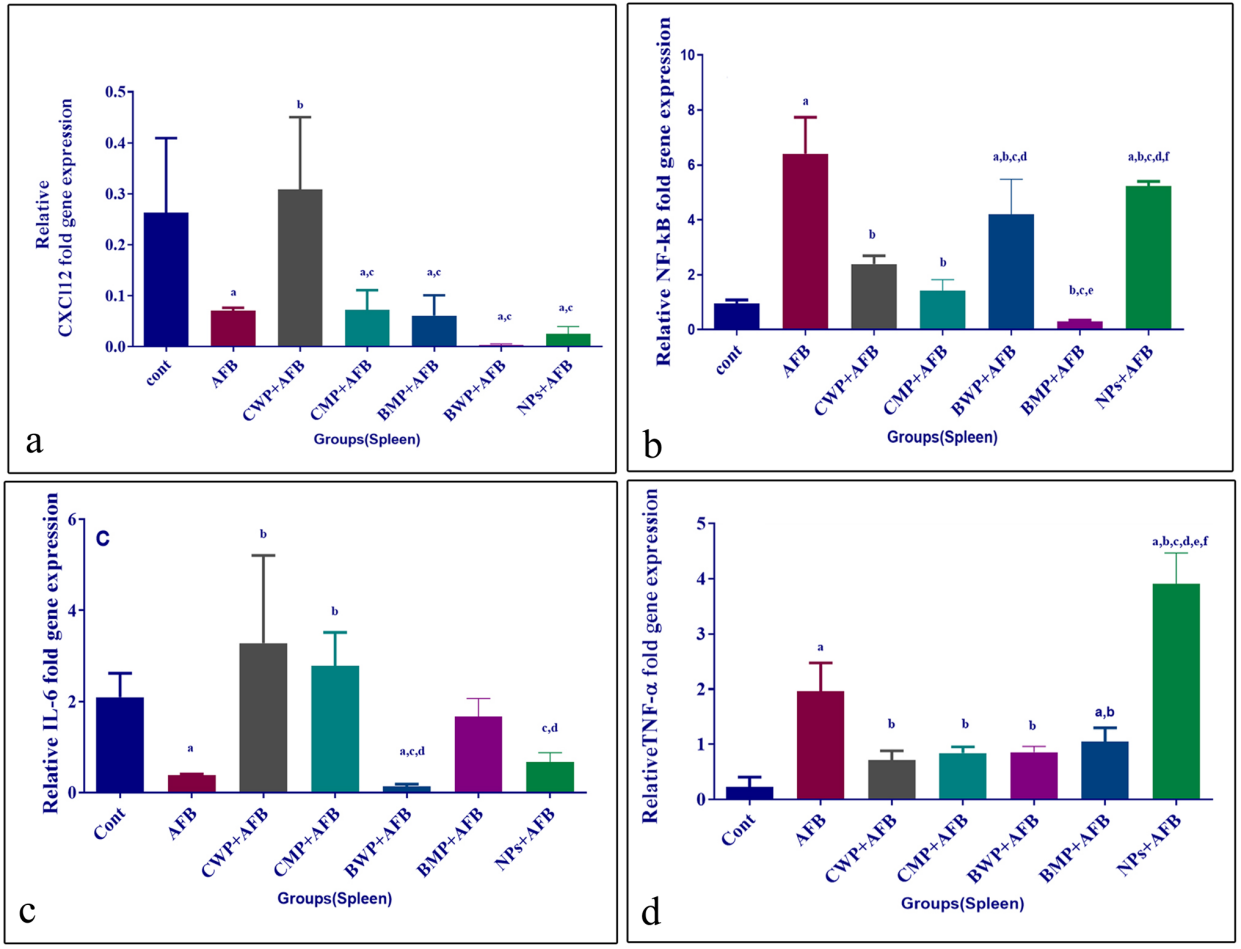
Effects of AFB intoxication and different whey proteins prophylactic treatments on the relative expression of CXCL12 (**A**), NF-κβ (**B**), IL-6 (**C**), and TNF-α (**D**) in the spleen. GAPDH is a control gene. Data are presented as means ± SEM. (n = 10). a, b, c, d, e, f significance difference from CONT group, AFB, CWP + AFB, CMP+AFB, BWP+AFB BMP+AFB, and NP+AFB-treated groups. The one-way analysis of variance with LSD post hoc test was used for analysis of the significance between groups. *AFB* aflatoxin B, *BMP* bovine whey protein PLGA-chitosan microparticles, *CMP* camel whey protein PLGA chitosan microparticles, *CONT* control group, *CXCL12* chemokine-x-C 12, *IL-6* interleukin-6, *NF-κβ* nuclear factor-kappaβ, *NPs* PLGA-chitosan nanoparticles, *TNF-α* Tumor necrosis factor-α

When compared to the aflatoxicated group, the CWP treatment significantly increased the expression of CXCL12 in the spleen, while other treatments failed to produce significantly lower levels (Fig. 3A). The expression levels of NF-κB and TNF-α were significantly increased in the AFB-intoxicated group in comparison to the reference control group (both p < 0.01). The four supplements reduced the NF-κB and TNF-α expression in comparison to the AFB-treated group, whereas, the AFB+BMP-treated group showed lower significant levels of NF-κβ expression than the AFB+BWP and AFB+CWP-treated groups (p < 0.001, p < 0.01, respectively); however, the AFB+BMP-treated group was the least supplemented to reduce the TNF-α levels in comparison to the AFB-intoxicated group **(**Fig. 3B, D). The group treated with AFB had a lower significant level of IL-6 in the spleen when compared to the reference control group (p < 0.05). The CWP and CMP treatments significantly increased the IL-6 levels in comparison to the AFB-intoxicated group. Neither the BWP nor BMP treatment increased IL-6 levels when compared to the AFB-intoxicated group (Fig. 3C).

### The effect of AFB and whey proteins on cleaved caspase-3 immunoblotting in the spleen and thymus

**Fig. 4:**
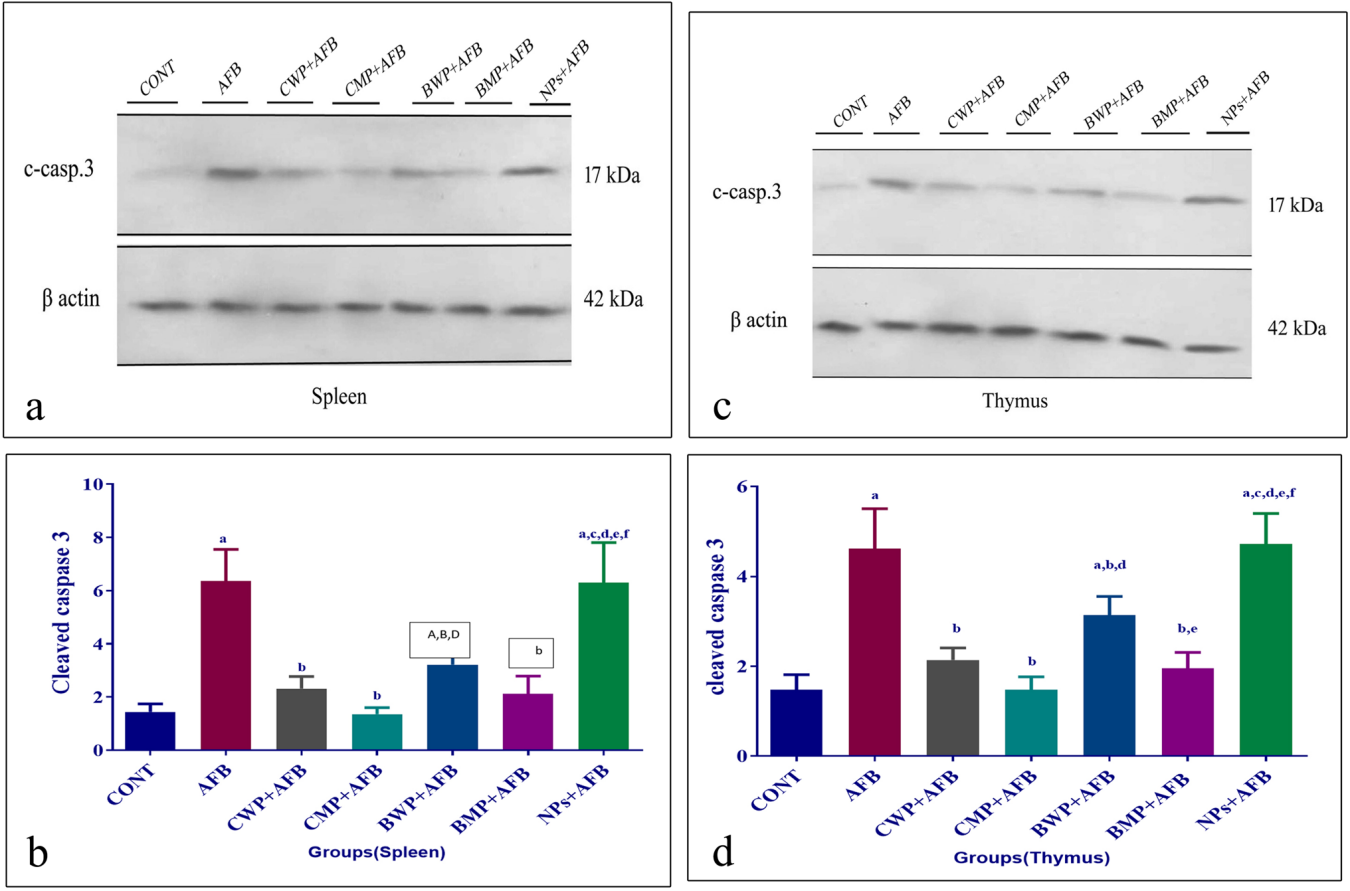
Effects of AFB intoxication and different whey proteins prophylactic treatments on the protein expression of cleaved caspase-3 by western blot in the spleen (**A**, **B**), thymus (**C**, **D**). B-actin is a control. Data are presented as means ± SEM. (n = 10). a, b, c, d, e, f significance difference from CONT group, AFB, CWP+AFB, CMP+AFB, BWP+AFB BMP+AFB, and NP+AFB-treated groups. The one-way analysis of variance with LSD post hoc test was used for analysis of the significance between groups. *AFB* aflatoxin B, *BMP* bovine whey protein PLGA-chitosan microparticles, *caspase-3* cysteine-aspartic acid protease-3 pase-3, *CMP* camel whey protein PLGA chitosan microparticles, *CONT* control group, *NPs* PLGA-chitosan nanoparticles

Cleaved caspase-3 relative protein expression was increased by aflatoxin B in both the thymus and spleen. All of the CWP, CMP, BWP, and BMP supplements reduced their levels significantly. In comparison to CWP and BWP treatments, CMP significantly reduced splenic and thymic cleaved caspase-3 levels (Fig. 4A–D).

### Histopathological findings

#### Histopathological examinations of the spleen

**Fig. 5:**
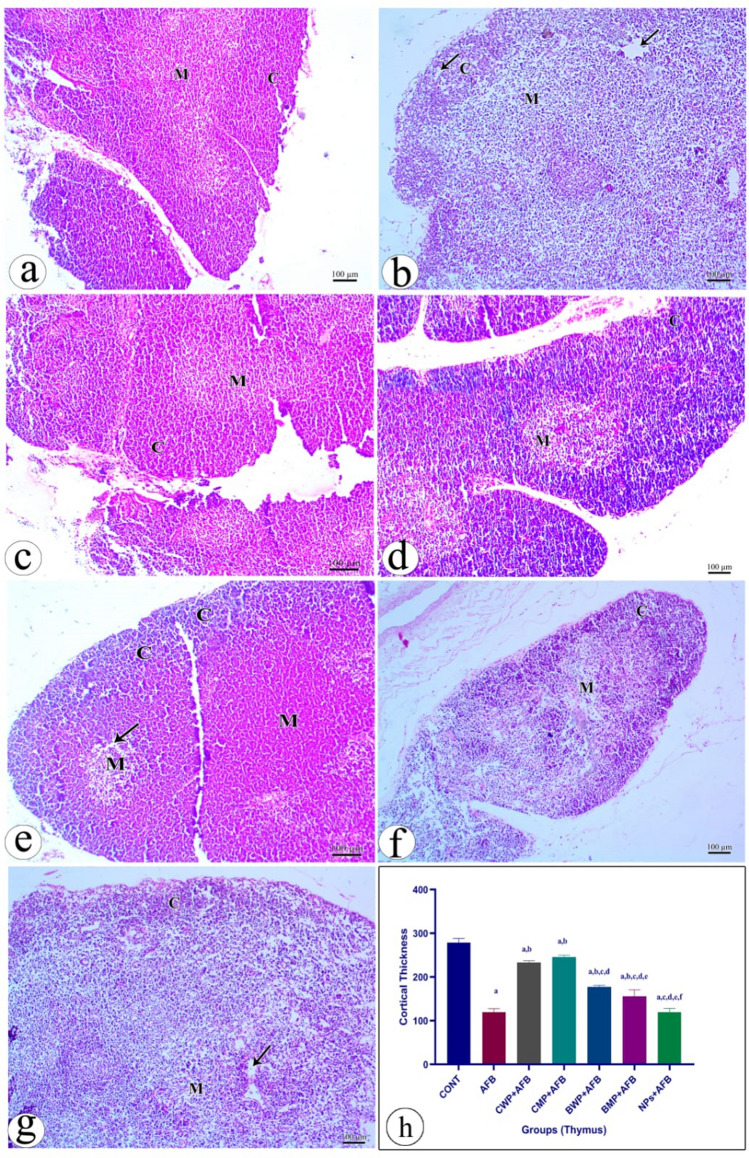
Photomicrographs of rat spleen stained with H&E stain presenting: **a** Section from the (control) group showed the parenchyma of the spleen that composed of white pulp macrophages (WP), red pulp macrophages (RP), note Central artery (Ca) was found. **b** Section from the (AFB) group presented parenchyma of the spleen (WP and RP) distortion in the organization of the histological structure: Shrinkage of the lymphatic nodules of white pulp and decreased cellularity in the follicle and marginal zone could be detected. Note the degeneration of some cells (arrow). **c** Section from the (CWP+AFB) group presented splenic pulp (WP and PR) showing the normal organization of the structure of white pulp (WP) and red pulp (RP). **d** Section from the (CMP+AFB) group showed normal organization of the structure of white pulp (WP) and red pulp (RP) (arrow). **e** Section from (BWP+AFB) group showed vacuolation of splenic cells, degeneration of lymphocytes in the white pulp was also observed. **f** section from the (BMP+AFB) group showed little cell degeneration with some disfigurement especially in RP. **g** Section from the (NPs+AFB) group showed vacuolated cells.** h** Bar chart show circumference of white pulp in different groups. Data are presented as means±SEM. (n = 10). a, b, c, d, e, f significance difference from CONT group, AFB, CWP+AFB, CMP+AFB, BWP+AFB BMP+AFB, and NP+AFB-treated groups. *AFB* aflatoxin B, *BMP* bovine whey protein PLGA-chitosan microparticles, *NPs* PLGA-chitosan nanoparticles. The one-way analysis of variance with LSD post hoc test was used for analysis of the significance between groups

Examination of spleen sections from the control group showed that the spleen is composed of white and red pulp. The white pulp consists of the central artery surrounded by the periarterial lymphoid sheath, with a normal distribution of lymphocytes in the lymphatic nodule. In between the white pulp is the red pulp, which is composed of splenic cords from Billroth and sinusoids. The red pulp (RP), which is composed of splenic cords and sinusoids, both of which contain blood cells of all types, splenic cords that are highly cellular contain plasma cells, white blood cells, and lymphocytes. The splenic sinusoids contain blood cells of all types (Fig. 5a). In the aflatoxin B-treated group, loss of architecture with shrinkage of the lymphatic nodules in white pulp and decreased cellularity in the follicle and marginal zone could be detected, and wide empty spaces among the cells in both white pulp and red pulp. Most cells showed degenerative changes, and many cells had pale vacuolated cytoplasm (Fig. 5b). In the CWP-treated group, there was a significant improvement in splenic tissues in the form of normal organisation of the structure of white pulp and red pulp, which was nearly identical to the control group. Also, white pulp and red pulp showed a normal appearance of the cells (Fig. 5c). The CMP-treated group showed normal organisation of the structure of white pulp and red pulp (Fig. 5d). In the BWP-treated group, there is a slight improvement, but still there is vacuolation of splenic cells and degeneration of lymphocytes in the white and red pulps (Fig. 5e). The BMP-treated group showed little cell degeneration with some disfigurement, especially in red pulp (Fig. 5f). A section from the NPs-treated group showed vacuolated cells. The bar chart shows the white pulp circumference in different treated groups. The AFB-intoxicated group had a significantly smaller WP circumference than the control group. The CWP, CMP, BWP, and BMP-treated group showed significant increases in white pulp circumference when compared to the AFB-treated group. The CWP and CMP treated-groups had significantly larger white pulp circumferences than the BWP and BMP-treated groups (Fig. 5h). Supplementary Fig. 3 shows magnified spleen sections from all groups.

#### Histopathological examinations of thymic tissue

**Fig. 6:**
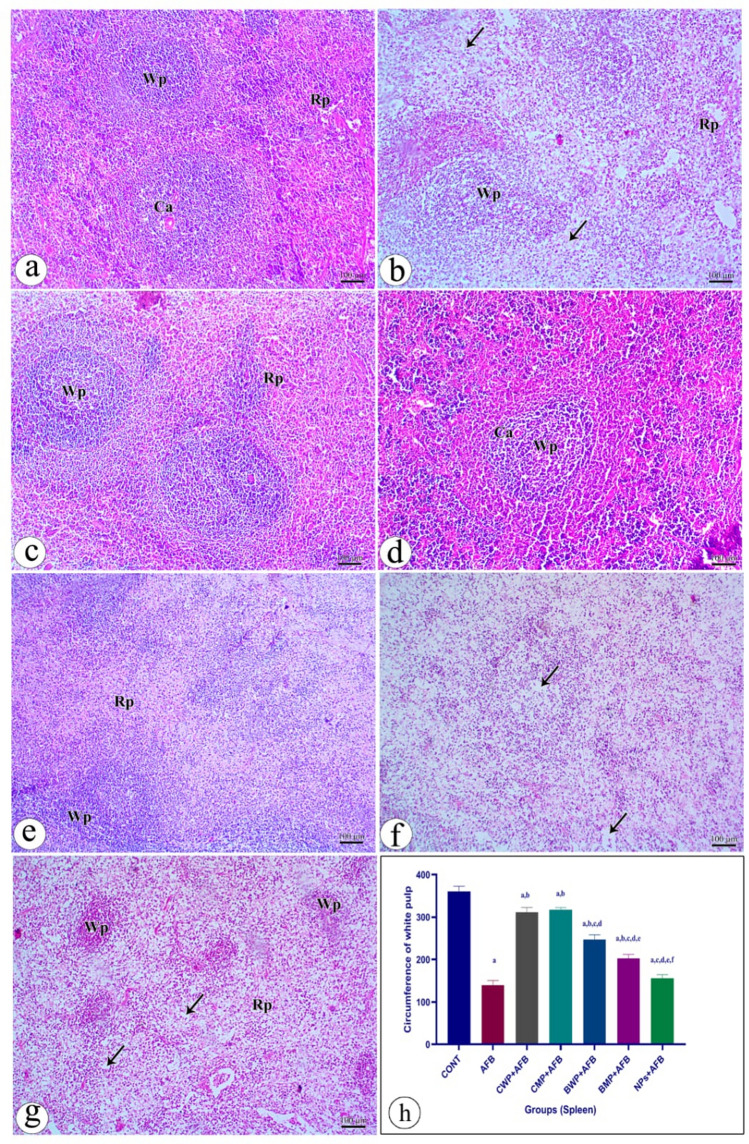
Photomicrographs of rat thymus stained with H&E stain presenting: **a** Section from the (control) group shows thymic lobules with normal architectures in form of outer darkly stained cortex and slightly paler medulla. **b** Section from the (AFB) group showed disfigurement in the organization with apparent decrease in the thickness of the cortex (cortical atrophy) with loss of corticomedullary demarcation, **c** Section from the (CWP+AFB) group shows slightly normal organization of the thymolobules with increase of the cortical thickness, **d** section from (CMP+AFB) group showed thymic lobules with normal architectures in form of outer darkly stained cortex and slightly paler medulla, **e** section from (BWP+AFB) atrophy slight demarcation appear between cortex and medulla, **f** section from (BMP+AFB) group showed lymphoid depletion with spaced between cells which more apparent in medulla, **g** section from (NPs+AFB) group showed space appeared between cells with a lot of vacuolated cells, **h** The effect of treatments on the thickness of the thymus cortical layer was displayed in a bar chart, which demonstrated that the CWP and CMP greatly raised the cortical thickness more than the BWP and BMP (Fig. 8H). Data are presented as means ± SEM. (n = 10).  a, b, c, d, e, f significance difference from CONT group, AFB, CWP+AFB, CMP+AFB, BWP+AFB BMP+AFB, and NP+AFB-treated groups. *AFB* aflatoxin B, *BMP* bovine whey protein PLGA-chitosan microparticles, *NPs* PLGA-chitosan nanoparticles. The one-way analysis of variance with LSD post hoc test was used for analysis of the significance between groups

Thymus sections from the control group were examined, and it was observed that the thymus is made up of two separate lobes joined by an isthmus of connective tissue. Each lobe is encased in a thin connective tissue capsule, which develops into septae that partially partition the thymus into lobules of varying sizes and orientations. An external, darkly stained cortex and a slightly paler medulla make up each thymic lobule. The medulla is pale coloured and less densely cellular than the cortex, which is made up of tightly packed tiny lymphocytes and few epithelial reticular cells. Large lymphocytes and a significant number of epithelial reticular cells were seen (Fig. 6a). Supplementary Fig. 4 shows magnified thymus sections from all groups.

In the AFB-intoxicated group, disfigurement was evident in the organization, with an apparent decrease in the thickness of the cortex (cortical atrophy), loss of corticomedullary demarcation, and wide spaces between cells with a lot of vacuolated cells (Fig. 6b). The CWP-treated group showed slightly normal organization of the thymus lobules with an increase in cortical thickness and most of the cells retaining their normal appearance with slight vacuolated cells still present (Fig. 6c). The CMP+AFB-treated group showed thymic lobules with normal architecture in the form of an outer, darkly stained cortex and a slightly paler medulla (Fig. 6d). The group that received BWP nevertheless had atrophy in the cortex, a minor demarcation between the cortex and medulla, and a slight lymphoid depletion with cell gaps that were more noticeable in the medulla (Fig. 6e). The BMP+AFB-treated group showed lymphoid depletion with space between cells that was more abundant in the medulla (Fig. 6f). The NPs-treated group showed space appeared between cells with a lot of vacuolated cells (Fig. 6g). A bar chart representing the impact of treatments on thymus cortical thickness revealed that the CWP and CMP considerably enhanced cortical thickness more than the BWP and BMP (Fig. 6h).

### Immunohistochemical staining of spleen and thymus

**Fig. 7:**
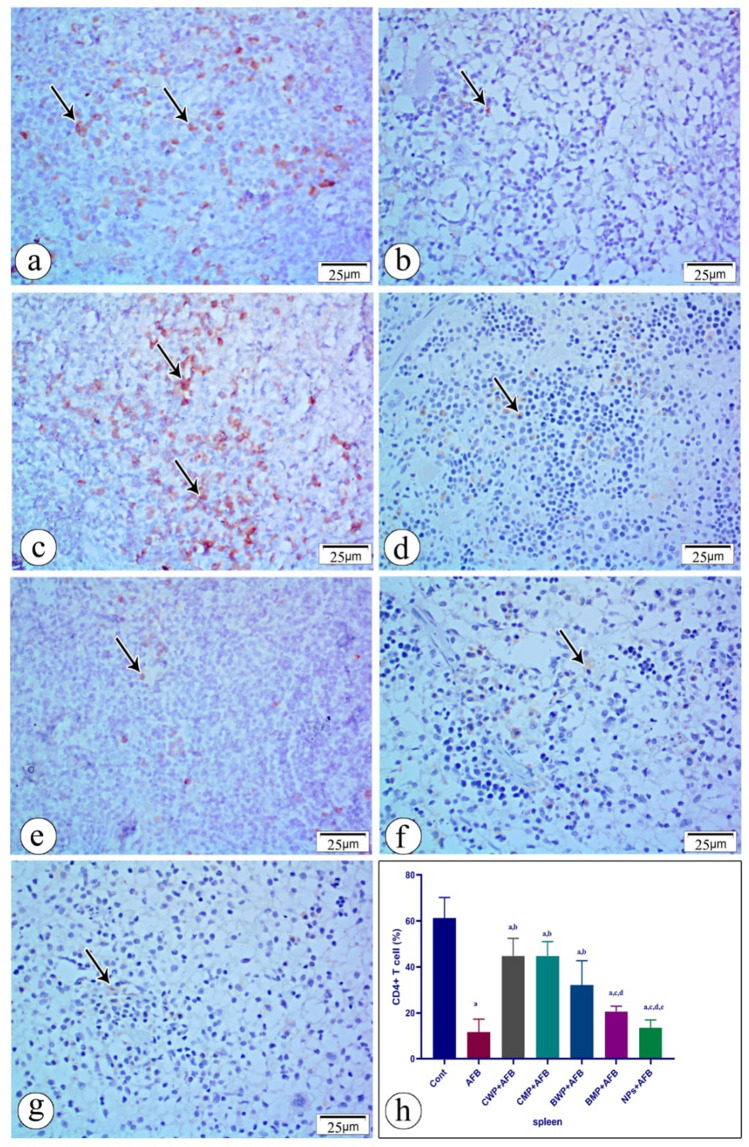
Immunostained sections with antibody CD4 showing its expression in rat spleen tissue in all studied groups. Positive staining is shown brown or red color **a** (CONT) group shows moderate immunoreactivity with anit-CD4 **b** AFB intoxicated group exhibit mild immunoreactivity with anti-CD4 in the splenic tissue section **c** (AFB+CWP) treated group shows moderate immunoreactivity with anti-CD 4 comparing to AFB intoxicated-group. **d** (AFB+CMP) treated group shows moderate immunoreactivity with anti- CD4 compared to the AFB-intoxicated group. **e** (AFB + BWP) treated group shows moderate immunoreactivity with anti-CD4 compared to the AFB-intoxicated group. **f** (AFB+BMP) treated group shows mild immunoreactivity with anti-CD4 compared to the AFB-intoxicated group. **g** (AFB+NPs) shows mild immunoreactivity with anti-CD4 compared to the AFB–intoxicated group. **h** Statistical analysis of the immunoreactivity with anti-CD4 in the seven groups. Data are presented as means±SEM (n = 10). a, b, c, d, e, f significance difference from CONT group, AFB, CWP+AFB, CMP+AFB, BWP+AFB BMP+AFB, and NP+AFB-treated groups. *AFB* aflatoxin B, *BMP* bovine whey protein PLGA-chitosan microparticles, *NPs* PLGA-chitosan nanoparticles. The one-way analysis of variance with LSD post hoc test was used for analysis of the significance between groups. (Color figure online)

**Fig. 8:**
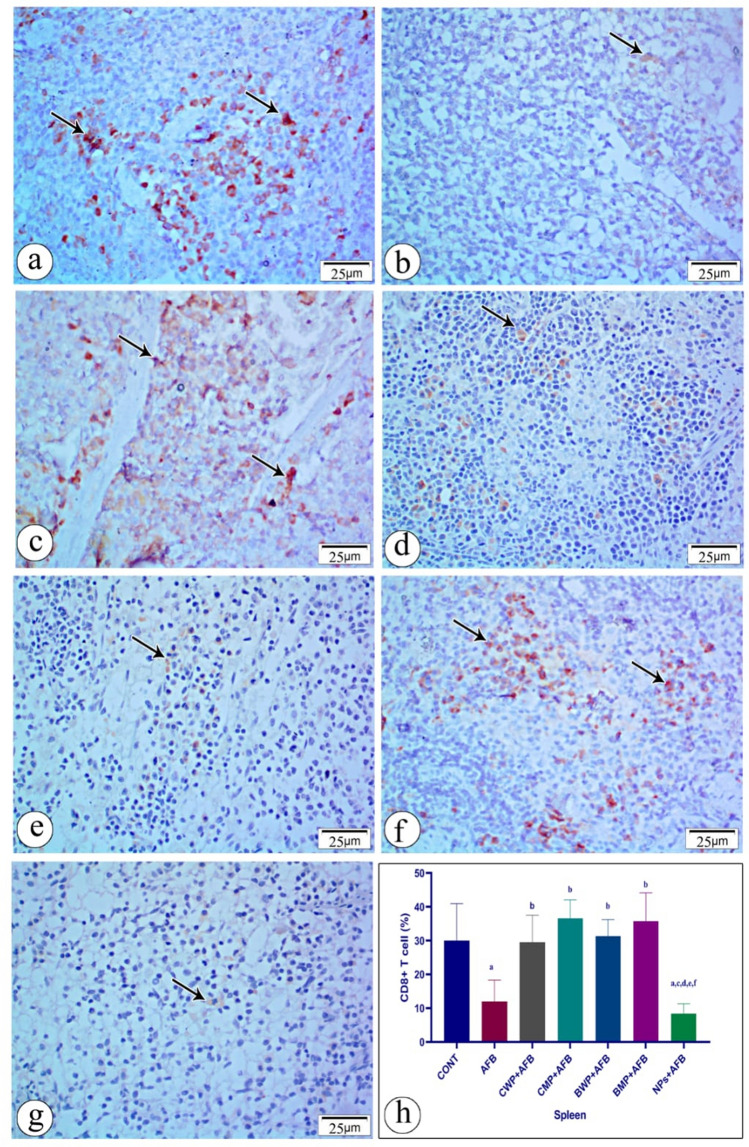
Immunostained sections with antibody CD8 showing its expression in rat spleen tissue in all studied groups. Positive staining is shown brown or red color **a **(CONT) group shows moderate immunoreactivity with anit-CD8. **b** AFB intoxicated group exhibit mild immunoreactivity with anti-CD8 in the splenic tissue section. **c** (AFB+CWP) treated group shows moderate immunoreactivity with anti-CD 8 comparing to AFB intoxicated-group. **d** (AFB + CMP) treated group shows moderate immunoreactivity with anti-CD8 compared to the AFB-intoxicated group. **e** (AFB+BWP) treated group shows moderate immunoreactivity with anti-CD8 compared to the AFB-intoxicated group. **f** (AFB+BMP) treated group shows moderate immunoreactivity with anti-CD8 compared to the AFB-intoxicated group. **g** (AFB+NPs) shows mild immunoreactivity with anti-CD8 compared to the AFB–intoxicated group. **h** Statistical analysis of the immunoreactivity with anti-CD8 in the seven groups. Data are presented as means ± SEM. (n = 10). a, b, c, d, e, f significance difference from CONT group, AFB, CWP+AFB, CMP+AFB, BWP+AFB BMP+AFB, and NP+AFB-treated groups. *AFB* aflatoxin B, *BMP* bovine whey protein PLGA-chitosan microparticles, *NPs* PLGA-chitosan nanoparticles. The one-way analysis of variance with LSD post hoc test was used for analysis of the significance between groups. (Color figure online)

Examination of anti-CD4 and anti-CD8 T lymphocyte immunostained splenic sections of the control reference group showed CD4 T and CD8T lymphocytes with moderate positive immunoreactivity, while the AFB-intoxicated group revealed few CD4 T and CD8 T lymphocytes with mild positive immunoreactivity. When compared to the AFB-intoxicated and NP-treated groups, the CWP, CMP, BWP, and BMP-treated groups displayed a larger proportion of CD8-positive immunostained cells. Compared to the AFB-intoxicated group, the CD4 T-cell count was greater with CWP, CMP, and BWP; however, there was no discernible difference between the BMP-treated group and the AFB-intoxicated group (Figs. 7 and 8).

**Fig. 9:**
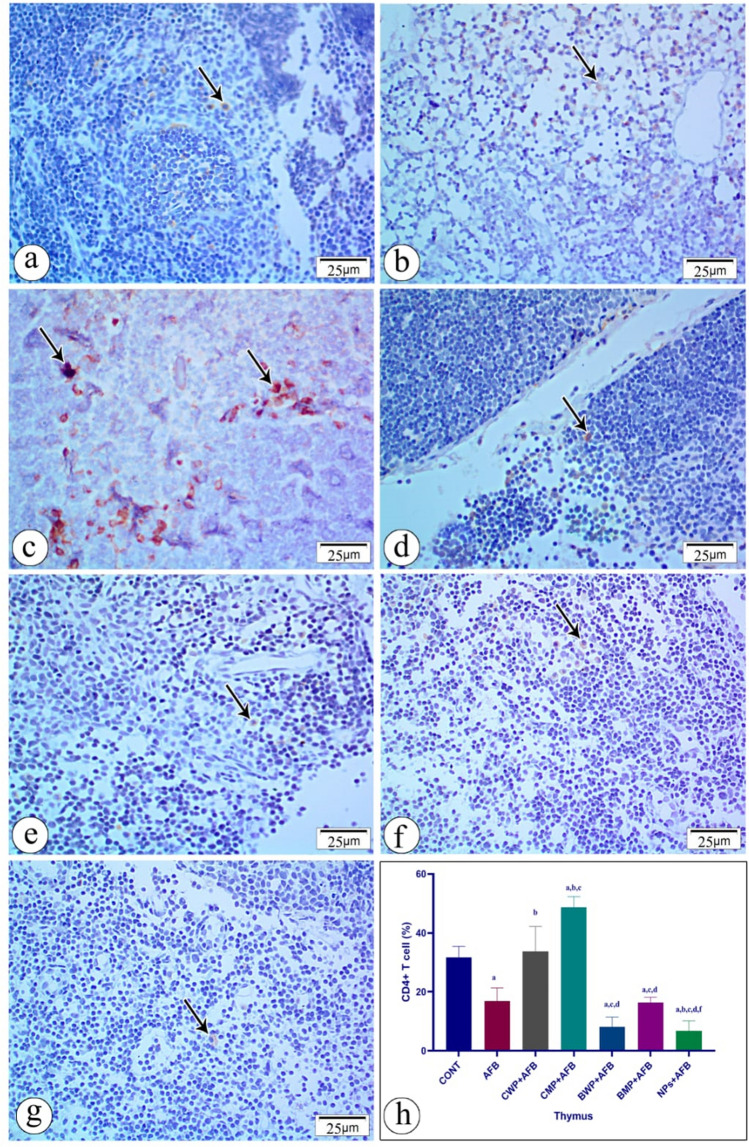
Immunostained sections with antibody CD4 showing its expression in rat thymus tissue in all studied groups. Positive staining is shown brown or red color **a ** (CONT) group shows moderate immunoreactivity with anit-CD4 **b** AFB intoxicated group exhibit mild immunoreactivity with anti-CD4 in the splenic tissue section **c** (AFB+CWP) treated group shows moderate immunoreactivity with anti-CD 4 comparing to AFB intoxicated-group. **d ** (AFB + CMP) treated group shows moderate immunoreactivity with anti- CD4 compared to the AFB-intoxicated group. **e** (AFB+BWP) treated group shows mild immunoreactivity with anti- CD4 compared to the AFB-intoxicated group. **f** (AFB+BMP) treated group shows mild immunoreactivity with anti- CD4 compared to the AFB-intoxicated group. **g** (AFB+NPs) shows mild immunoreactivity with anti- CD4 compared to the AFB--intoxicated group. **h** Statistical analysis of the immunoreactivity with anti-CD4 in the seven groups. Data are presented as means ± SEM. (n=10). a, b, c, d, e, f significance difference from CONT group, AFB, CWP+AFB, CMP+AFB, BWP+AFB BMP+AFB, and NP+AFB-treated groups.* AFB* aflatoxin B,* BMP* bovine whey protein PLGA-chitosan microparticles,* NPs* PLGA-chitosan nanoparticles. The one‐way analysis of variance with LSD post hoc test was used for analysis of the significance between groups. (Color figure online)

**Fig. 10:**
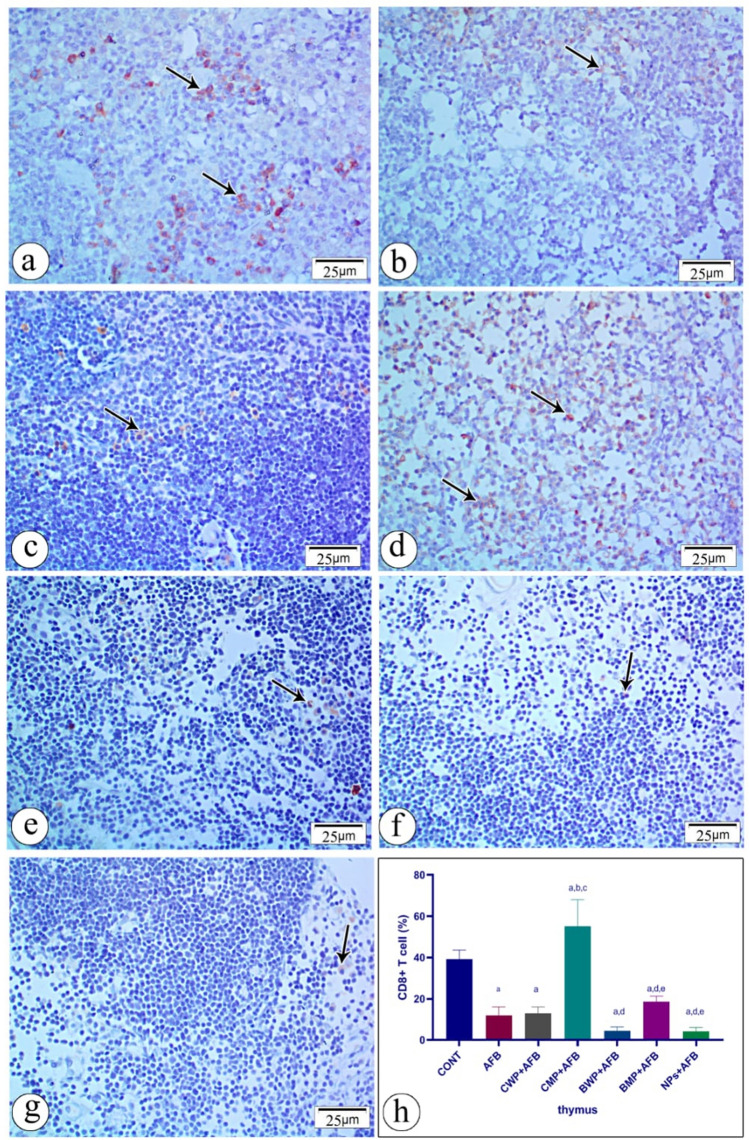
Immunostained sections with antibody CD8 showing its expression in rat thymus tissue in all studied groups. Positive staining is shown brown or red color **a **(CONT) group shows strong positive immunoreactivity with anit-CD8. **b** AFB intoxicated group exhibit mild immunoreactivity with anti-CD8 in the splenic tissue section. **c** (AFB+CWP) treated group shows mild immunoreactivity with anti-CD 8 comparing to AFB intoxicated-group. **d (**AFB+CMP) treated group shows strong positive immunoreactivity with anti-CD8 compared to the AFB-intoxicated group. **e** (AFB+BWP) treated group shows mild immunoreactivity with anti- CD8 compared to the AFB-intoxicated group. **f** (AFB+BMP) treated group shows mild immunoreactivity with anti-CD8 compared to the AFB-intoxicated group. **g** (AFB+NPs) shows mild immunoreactivity with anti- CD8 compared to the AFB–intoxicated group. **h** Statistical analysis of the immunoreactivity with anti-CD8 in the seven groups. Data are presented as means±SEM (n = 10). a, b, c, d, e, f significance difference from CONT group, AFB, CWP+AFB, CMP+AFB, BWP+AFB BMP+AFB, and NP+AFB-treated groups. *AFB* aflatoxin B, *BMP* bovine whey protein PLGA-chitosan microparticles, *NPs* PLGA-chitosan nanoparticles. The one-way analysis of variance with LSD post hoc test was used for analysis of the significance between groups. (Color figure online)

The effect of AFB intoxication and different supplements on CD4 and CD8 immunostaining in the thymus is shown in Figs. 9 and 10. Contrary to the control groups, aflatoxicosis led to a decrease in CD4 and CD8 immunostained cells, the CMP and CWP considerably raised the CD4 immunostained cells, and the CMP-treated group showed significantly more CD4 T lymphocytes than the CWP-treated group. Whereas C8 T cells were exclusively enhanced in the CMP-treated group. In comparison to the AFB-intoxicated group, there were no noteworthy variations in the percentages of CD4 T cells or CD8 T immunostained cells between the BWP, BMP, and NPs-treated groups.

## Discussion

The current study was concerned with the more protective effects of CWP or BWP acting against aflatoxicosis in rats, particularly on the liver and the immunological tissues of the spleen and thymus. Noting that the Native-PAGE electrophoretic pattern showed that the most prominent electrophoretic zone in CWP was the lactoferrin fraction, which constituted 33% of CWP, this is compatible with Ebiad et al. study [24], while the more constituting fraction in the pattern of BWP was for α-lactalbumin, which constitutes 27% of bovine whey proteins [25].

The metabolism of AFB in mammalian hepatic tissue results in oxidative stress and subsequently inflammation [34]. Our study showed remarkable higher hepatic MDA level, which agreed with that reported by Rotimi et al. [35] as subacute mitochondrial intoxication. One major consequence of lipid peroxidation is the release of NO which augment cellular damage [34].

Since the metabolism of AFB1 passes through two phases of metabolism, where in phase 2 reactions, the AFB metabolites had been detoxified by conjugation with reduced GSH via GST which leads to their depletion. This explains the observed decline in the levels of antioxidants in the liver [36].

Herein assayed antioxidant effects of CWP and CMP have been recorded as significantly reduced levels of oxidative stress and inversely increased antioxidant enzymes activities in taken liver tissues, agreed with what was reported by Du et al. study [37]. In this aspect, the CMP exhibited more powerful antioxidant activity than BWP. Whether prophylactic or for treatment, camel milk contains higher lactoferrin than bovine milk [38] and encapsulation of camel whey protein with active ingredients of microparticles can enhance their biological activity [39].

In this work, the reduced MDA levels in the tested livers of the treated BWP group were reasonable with those of others like Wang et al. [40] who reported that increasing dietary bovine lactoferrin led to decline in MAD by sequestering iron. In this aspect, bovine α-LA augment the antioxidant enzyme function due to increase of hepatic GSH [41] similar to the herein deduced results where BWP could restore oxidative balance.

Regardingly, chemokines play important role in splenic structure. Basically, our derived data demonstrated that shrinkage of splenic lymphoid follicles (with loss of organization of white pulp) presumably due to generation of free radicals and apoptosis [42] and a decline in CXCL12 level in AFB1 treated rats means that a decline in the migration of lymphocytes. This contributed to AFB1 induced immunosuppression. Some scientists reported previously that AFB-contaminated feed cause inhibition of T-cell multiplication [43].

Noting that the tested CWP treatment against aflatoxicosis showed an improvement in spleen structure. In this research, a significant increase in splenic mRNA NF-κB expression and decrease in CXCL12 in aflatoxicosed animals as there is an inverse relationship between them. The results could be explained according to Madge and May [44] who revealed NF-κB upregulation associated with down regulation of CXCL12 during AFB intoxication as higher NF-κB levels is crucial for immune cells tasks and growth after AFB exposure, the stimulatory effect of AFB on NF-κB [45]. Simultaneously the current study showed an obvious lower number of CD4 and CD8 subsets than in those in healthy group.

Assay of splenic expression of TNF-α in the spleen was also significantly up-regulated in aflatoxicosed rats in comparison to the control group. Our results agreed with Helmy et al. [46] who reported that aflatoxins had a serious effect on the tested immune system in albino female mice as it promoted pro-inflammatory cytokine TNF-α through the oxidative stress-mediated mechanism. The results contradict the findings of another study in which a decrease in serum and expression of TNF-α within splenocytes during aflatoxicosis [47].

Since IL-6 is an immunomodulatory and inflammatory cytokine, AFBI exerts its immune-toxic effects by altering IL-6 production. Our results recorded an obvious decline in IL-6 levels in the spleen organic tissue of aflatoxicosed rats, these findings agreed with He et al. [48] who recorded that expressed mRNA contents of IL-6 in the AFB1 group in the ileum was decreased, as a consequence of the decline in mature T lymphocytes and secreted fewer cytokines, also the decreased content of IL-6 can reduce the proliferation of T lymphocytes.

Aflatoxicosis directly or indirectly activates the apoptotic process as Zhu et al. (2017) study [49] has demonstrated that the AFB in diet led to the elevated expression of TNF-R1, and caspase-3 mRNA expression in the spleen as it was assayed in this work. Therefore, it is tempting to speculate that the increased apoptosis of splenocytes provoked by AFB1 might lead to lymphocyte depletion, which may partly be responsible for immunosuppression in various circumstances.

Thymocyte depletion was observed in several infectious diseases as well as changes in the migratory responses. As mentioned earlier, thymocyte depletion is accompanied by a decline of chemokines CXCL12 by Mendes s-da‐Cruz et al. study [50] which agreed with our results as we recorded a decrease in CXCL12 level in the thymus of aflatoxicosed rats.

In this aspect, [51] have reported that CXCL12 may regulate or induce the expression of IL-6, which is an important key regulator of CXCL12 upon which it can explain our results as the level of IL-6 expression in the thymus increased inversely with the decrease of CXCL12 in the thymus. Such dysregulated IL-6 expression in aflatoxicated animals is indicative of tissue damage and inflammatory reactions by aflatoxin metabolites as well as a predictive of autoimmunity [48]. These current results about the elevation of IL-6 and TNF-α expression in the thymus by aflatoxicosis may be attributed to the increased oxidative stress that depleted the GSH, GST, and GPX and the increased NO and MDA [52].

Since a high dose of AFB enhanced leukocyte apoptosis with concurrent immune suppression [53], increased NF-κB nuclear translocation in T cells was stimulated by oxidative stress. In this aspect of cell signalling, NF-κB-induced TNF-α-mediated immune cell apoptosis in the thymus [4].

TNF-α has roles in cell death-caspase-related cascade and its opposite cell survival/NF-κB pathway [54]. Stimulationof apoptosis results in suppression of NF-κB/cell survival pathway. The collaborating cellular signals explain our results, as we have recorded significant induction of NF-κB with increasing TNF-α in the thymus in aflatoxicosed rats.

Our deduction can be explained according to Ibrahim et al. [52], who attributed the anti-inflammatory effect of camel^’^s milk to the decrease of NF-κB in breast cancer. CWP alleviated heat stress-induced lymphocyte depression by inhibiting the activation of NF-κB [53]. This also agreed with our results, as we have reported a reduction of NF-κB accompanied by TNF-α down-regulation in the CWP-treated group in immune tissues.

Furthermore, a previous study reported that the anti-inflammatory effect of camel’s whey protein on diabetic rats can be due to inhibiting the expression of circulatory TNF-α and thymic IL-6 [55]. Our findings about the NF-κB and TNF-α decrease in the thymus and spleen by CWP can be considered an acute phase of the inflammatory response.

Since B-cell apoptosis is related to inflammatory cytokines activation [56]. Basically, CWP resulted in a significant reduction in lymphocyte apoptosis which we have recorded herein as a significant reduction in cleaved caspase-3 in the CWP-treated group.

The herein observed decrease in NO levels in the AFB+BWP-treated group may be attributed to the to the direct scavenging effect of α-LA as it was stated by [57]. Moreover, blockade of endogenous nitric oxide (NO) synthesis and suppression of the generation of ROS blocking the activation of NF-κB was denoted by Yan et al.study [58].

The presented findings suggest that α-LA suppresses AFB-induced IL-6 release by inhibiting NF-κB. This explanation clarified our results, which showed suppression of hepatic NO in intoxicated rats treated with BWP correlated with suppression of NF-κB in the thymus and spleen compared to the reduction of IL-6 in the thymus as well as a reduction in TNF-α in the thymus and spleen. Noticeably, the interaction between bovine α-LA and macrophages resulting in suppression of inflammation has verified the role of BWP in suppressing IL-6 in the thymus due to its anti-inflammatory characteristics, as proved by Park et al. [59].

Hence, the BWP decreased NO in intoxicated liver of rats as previously confirmed by Qiugang et al. [53] study, which reported suppression of NO and iNOS through affection of the NF-κB. However, α-LA control iNOS mRNA expression by activation of cytokines; amelioration of liver injury due to AFB1 was associated with NF-κB/iNOS/NO pathway suppression by α-LA supplementation. The feature that we have recorded in this work is the inhibition of NF-κB mRNA expression in immune tissues associated with hepatic NO suppression.

Some bovine whey proteins, such β-LG and α-LA, remain intact after absorption [60]. A high-whey proteins has also been demonstrated to have similar immunomodulatory, anti-inflammatory, and antioxidative properties in both its undigested and digested forms [61]. In this regard, it may be hypothesized that such intact whey protein may be able to get contact with immune cells.

Anyhow, our provided results are consistent with those of Kanekanian [62], who has proven α-LA-induced anti-inflammatory roles. Taking into consideration α-LA reduced the amount of NF-kB that is expressed as a result of AFB1. The hepatoprotective benefits of α-LA against liver damage brought on by AFB1 are also caused by a lower generation of IL-6 and inhibition of inflammatory reactions.

High doses of whey proteins and lactoferrin dramatically decreased the alteration in hepatic architecture and hepatocyte apoptosis, inhibited the release of TNF-α, and strengthened the hepatic antioxidant defence system [63]. To control the TNF-α-induced extrinsic apoptotic pathway, cytosol GSH suppressed caspase-3 activity and turned on the hepatocytes’ nuclear factor-kB (NF-κB)-dependent survival pathways [64]. WPC significantly decreased activated caspase-3 as a result of increased GSH [65]. This result was consistent with our findings. These data revealed that camel whey proteins are more significant in protecting the liver, spleen, and thymus from the hazardous effects of aflatoxins than bovine whey proteins.

## Conclusions

The present Work provides convincing evidence for the immunomodulatory functions of CWP, CPM, and BMP in improving the effectiveness of chemotaxis of various immune cells towards various chemokines. We have focused on the importance of the antioxidant role of whey proteins. Our research proves that CWP increased CXCL12 in the spleen and thymus tissues which means improvement in the chemotaxis of B-cell and T-cell migration in the immune organs. So, CWP treatment enhances innate immunity by improving B and T cell chemotaxis efficiency in aflatoxicosis rats. The current work thus offers up opportunities for further investigation into the combination of antioxidants and CXCL12 agonists as a means of reducing the harmful ROS effects linked to aflatoxicosis.

The relatively low cost, safety, and efficacy via multiple molecular targets of camel whey protein and bovine whey proteins offer advantages of them over the chemical treatments. Accordingly, we recommend the use of this natural supplement in a daily diet. Moreover, these proteins deserve further investigation as adjuvant therapies in animals and man.

### Supplementary Information

Below is the link to the electronic supplementary material.

Supplementary Fig. 1: The SDS-PAGE pattern of whey proteins in camel milk **A** and bovine milk **B**. *Lane 1* molecular weight marker, *lane 2 and 3* whey samples, *LF* lactoferrin, *α-LA* α-lactalbumin (JPG 368.2 kb)

Supplementary Fig. 2:  Analysis study of CMP and BMP. **A** Size distribution of CMP (camel whey proteins PLGA-chitosan microparticles) shows CMPs size distribution by intensity is 1046 nm = 1um; PDI = 0.1. **B** TEM image for CMP shows spherical MPs with a micro-capsule of PLGA and Chitosan **C** Zeta potential of CMP is + 8.13 mV. **D** size distribution of BMP (bovine whey proteins PLGA-chitosan microparticles) shows BMP size distribution is 1040 nm = 1 um; PDI= 0.1. **E** TEM image for BMPs (E) as the image shows spherical MPs with a micro-capsule of PLGA and Chitosan. **F** Zeta potential of BMPs is − 16.7 mV (JPG 1186.1 kb)

Supplementary Fig 3: Photomicrographs of rat spleen stained with H&E stain showing: **a** Sections from the (control) group presented the (WP), part of (RP) and central artery (Ca). **b** Section from the (Aflatoxin) group presented RP loss of their architecture and congestion and there were cells with vacuolated pale cytoplasm. **c** Sections from the (CWP+AFB) group presented white pulp macrophages (WP) with slight iron loading (head arrow). Note: some cells are still vacuolated (star). **d** Section from the (CMP+AFB) group showed normal organization of the structure of white pulp (WP) and red pulp (RP). **e** Section from the (BWP+AFB) group showed vacuolation of splenic cells, degeneration of lymphocytes in the white pulpwas also observed. **f** Section from the (BMP+AFB) group showed the number of lymphocytes was lightly vacuolated (arrows) in lymphatic nodule and periarterial lymphatic sheath, as well as in the red pulp. **g** Section from the (NPs+AFB) group showed a lot of cells appeared vacuolated and degenerated (JPG 459.4 kb)

Supplementary Fig 4: Photomicrographs of rat Thymus stained with H&E stain showing: **a** Sections from the (control) group presented the cortex formed from densely packed small lymphocytes with few epithelial reticular cells (C) while medulla (M) is pale stained less densely cellular than cortex. It contained large lymphocytes and a lot of epithelial reticular cells. **b** Section from the (Aflatoxin) group presented wide space appeared between cells with a lot of vacuolated cells. **c** Sections from the (CWP+AFB) group presented shows slightly normal organization of the thymic lobules with increase of the cortical thickness. **d** Section from the (CMP+AFB) group showed most of the cells retaining their normal appearance with slight vacuolated cells still present. **e** Section from the (BWP+AFB) group showed cortex (C) and medulla (M).  Cortex still showing atrophy slight demarcation appear between cortex and medulla. **f** Section from the (BMP+AFB) group showed slight lymphoid depletion with spaced between cells which more apparent in medulla. **g** Section from the (NPs+AFB) group showed wide space appeared between cells with a lot of vacuolated cells (arrow). (JPG 367.0 kb)

Supplementary material 5 (DOCX 14.1 kb)

## Data Availability

The data will be available from the authors on reasonable request.

## Abbreviations

AFB1: Aflatoxin B1
BMP: Bovine whey protein-PLGA-chitosan microparticles
BWP: Bovine whey proteins
Caspase-3: Cysteine-aspartic acid protease-3
CMP: Camel whey protein-PLGA-chitosan microparticles
CWP: Camel whey proteins
CXCL12: C–X–C motif chemokine ligand 12
DLS: Dynamic light scattering
GST: Glutathione-S‐transferase
GSH: Reduced glutathione
GSH-Px: Glutathione peroxidase
iNOS: Inducible nitric oxide synthase
IL-6: Interleukin-6
LA: Lactalbumin
Lf: Lactoferrin
LG: Lactoglobulin
MDA: Malondialdehyde
MPs: Microparticles
NF-κB: Nuclear factor kappa B
NPs: Nanoparticles
NO: Nitric oxide
PLGA: Poly (d, l-lactide-co-glycolide)
ROS: Reactive oxygen species
SDF-1: Stromal cell-derived factor-1
TEM: Transition electron microscopy
TNF-α: Tumor necrosis factor-α
WPs: Whey proteins
