# Supplementary figures and images for: Antiapoptotic and chemotaxis-stimulating effects of poly (d, l-lactide-co-glycolide)-chitosan and whey proteins against aflatoxicosis-induced splenic and thymic atrophy
Source: Mol Biol Rep. 2023 Oct 15;50(12):9805–24. doi: 10.1007/s11033-023-08902-7 (PMC10676322; doi:10.1007/s11033-023-08902-7)

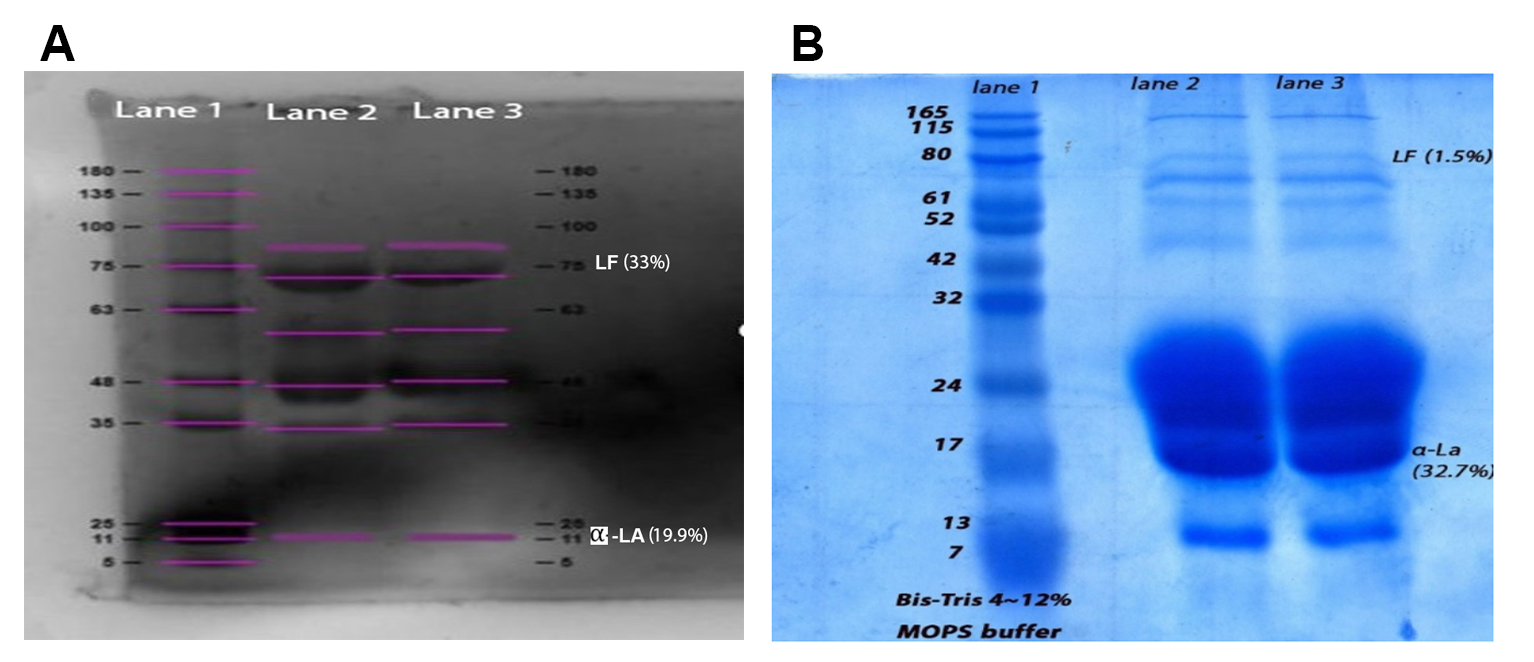

Supplement: Supplementary file 1 — Supplementary Fig. 1: The SDS-PAGE pattern of whey proteins in camel milk A and bovine milk B. Lane 1 molecular weight marker, lane 2 and 3 whey samples, LF lactoferrin, α-LA α-lactalbumin (JPG 368.2 kb) [file 11033_2023_8902_MOESM1_ESM.jpg]

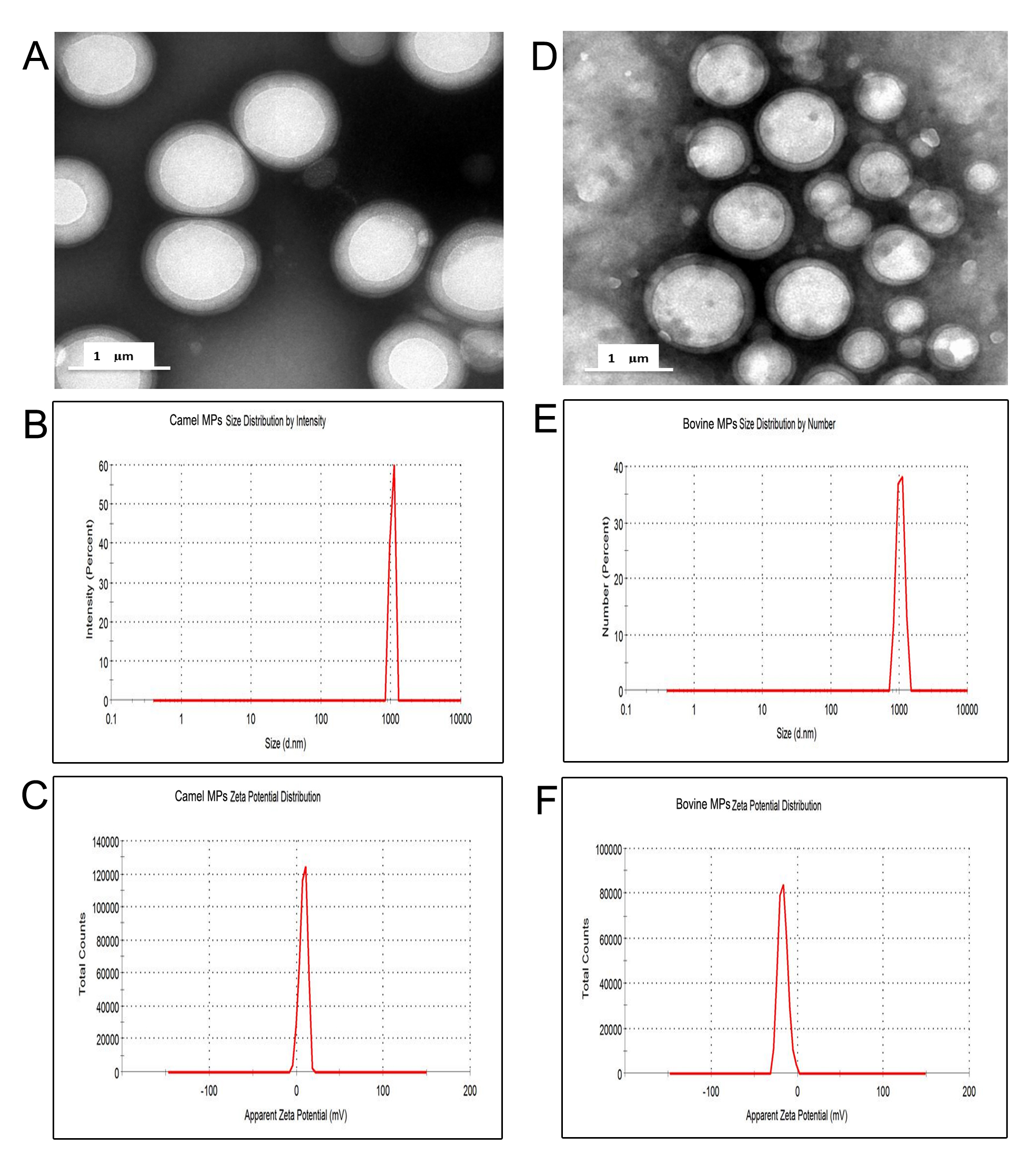

Supplement: Supplementary file 2 — Supplementary Fig. 2: Analysis study of CMP and BMP. A Size distribution of CMP (camel whey proteins PLGA-chitosan microparticles) shows CMPs size distribution by intensity is 1046 nm = 1um; PDI = 0.1. B TEM image for CMP shows spherical MPs with a micro-capsule of PLGA and Chitosan C Zeta potential of CMP is + 8.13 mV. D size distribution of BMP (bovine whey proteins PLGA-chitosan microparticles) shows BMP size distribution is 1040 nm = 1 um; PDI= 0.1. E TEM image for BMPs (E) as the image shows spherical MPs with a micro-capsule of PLGA and Chitosan. F Zeta potential of BMPs is − 16.7 mV (JPG 1186.1 kb) [file 11033_2023_8902_MOESM2_ESM.jpg]

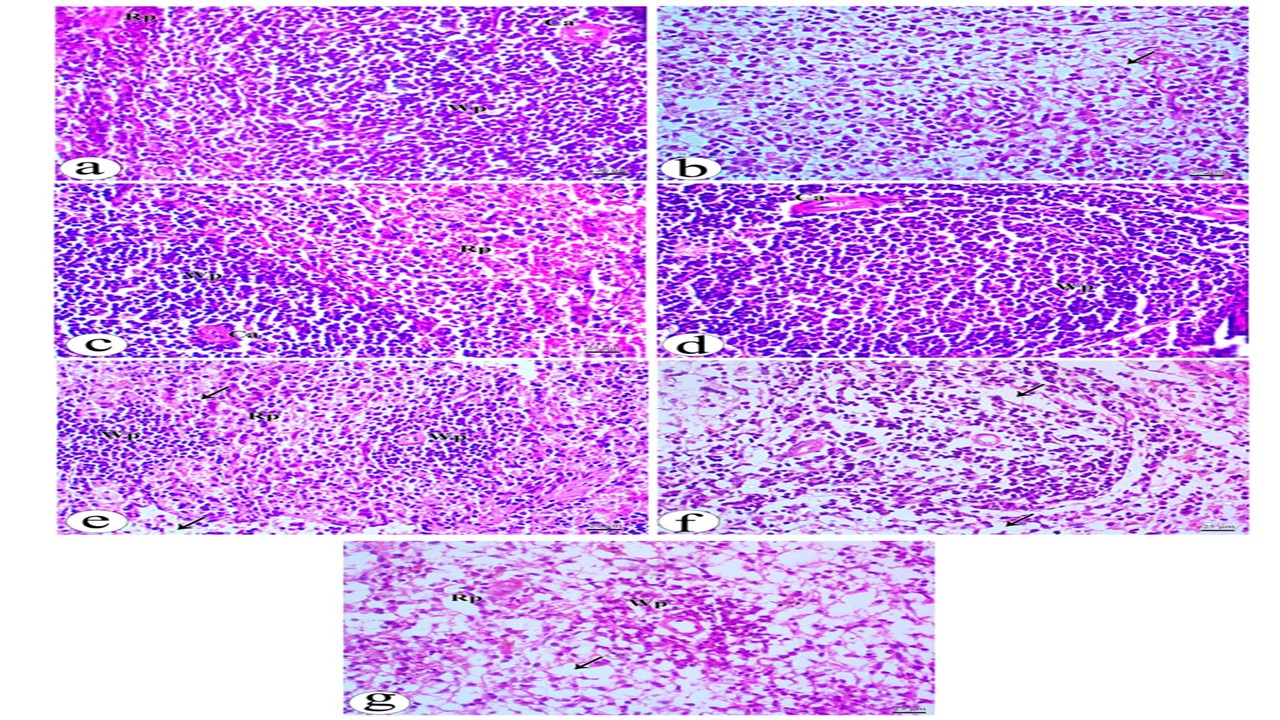

Supplement: Supplementary file 3 — Supplementary Fig 3: Photomicrographs of rat spleen stained with H&E stain showing: a Sections from the (control) group presented the (WP), part of (RP) and central artery (Ca). b Section from the (Aflatoxin) group presented RP loss of their architecture and congestion and there were cells with vacuolated pale cytoplasm. c Sections from the (CWP+AFB) group presented white pulp macrophages (WP) with slight iron loading (head arrow). Note: some cells are still vacuolated (star). d Section from the (CMP+AFB) group showed normal organization of the structure of white pulp (WP) and red pulp (RP). e Section from the (BWP+AFB) group showed vacuolation of splenic cells, degeneration of lymphocytes in the white pulpwas also observed. f Section from the (BMP+AFB) group showed the number of lymphocytes was lightly vacuolated (arrows) in lymphatic nodule and periarterial lymphatic sheath, as well as in the red pulp. g Section from the (NPs+AFB) group showed a lot of cells appeared vacuolated and degenerated (JPG 459.4 kb) [file 11033_2023_8902_MOESM3_ESM.jpg]

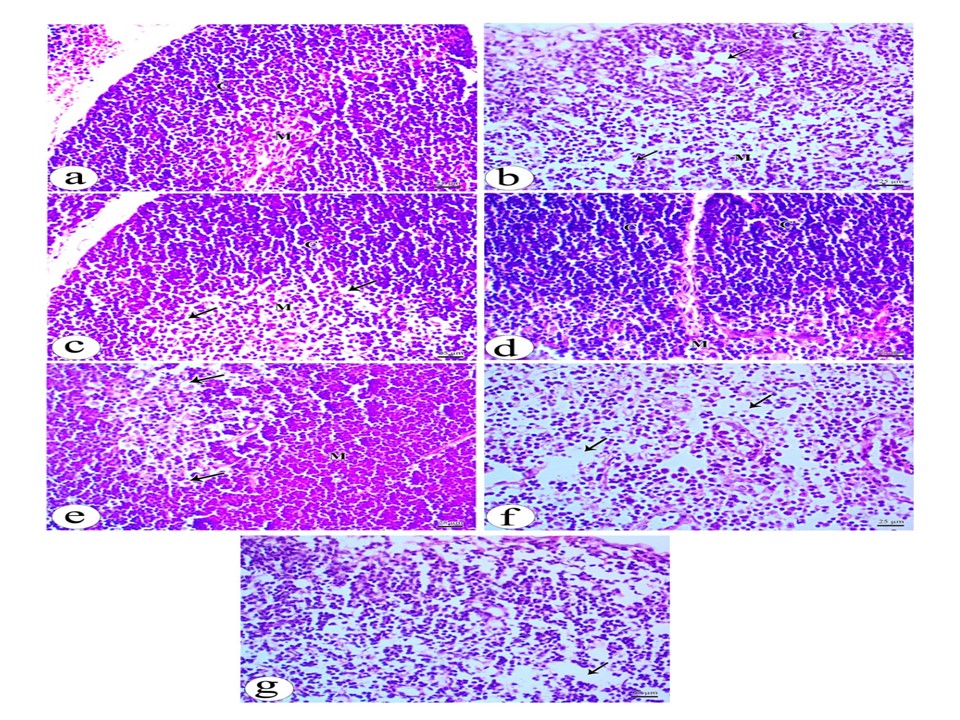

Supplement: Supplementary file 4 — Supplementary Fig 4: Photomicrographs of rat Thymus stained with H&E stain showing: a Sections from the (control) group presented the cortex formed from densely packed small lymphocytes with few epithelial reticular cells (C) while medulla (M) is pale stained less densely cellular than cortex. It contained large lymphocytes and a lot of epithelial reticular cells. b Section from the (Aflatoxin) group presented wide space appeared between cells with a lot of vacuolated cells. c Sections from the (CWP+AFB) group presented shows slightly normal organization of the thymic lobules with increase of the cortical thickness. d Section from the (CMP+AFB) group showed most of the cells retaining their normal appearance with slight vacuolated cells still present. e Section from the (BWP+AFB) group showed cortex (C) and medulla (M). Cortex still showing atrophy slight demarcation appear between cortex and medulla. f Section from the (BMP+AFB) group showed slight lymphoid depletion with spaced between cells which more apparent in medulla. g Section from the (NPs+AFB) group showed wide space appeared between cells with a lot of vacuolated cells (arrow). (JPG 367.0 kb) [file 11033_2023_8902_MOESM4_ESM.jpg]
