# Supplementary material for: Antiapoptotic and chemotaxis-stimulating effects of poly (d, l-lactide-co-glycolide)-chitosan and whey proteins against aflatoxicosis-induced splenic and thymic atrophy
Source: Mol Biol Rep. 2023 Oct 15;50(12):9805–24. doi: 10.1007/s11033-023-08902-7 (PMC10676322; doi:10.1007/s11033-023-08902-7)
Supplement: Supplementary file 5 — Supplementary material 5 (DOCX 14.1 kb) [file 11033_2023_8902_MOESM5_ESM.docx]

**Supplementary Table 1: Primer sequences of related genes that were used for RT-qPCR**

| **Primers** | **Primer sequences 5′-3′** |
| --- | --- |
| CXCL12  (SDF1) | Forward: TGAGGCCAGGGAAGAGTGAG  Reverse: GACACATGGCGATGAATGGA |
| IL-6 | Forward: TCTCTCCGGAAGAGACTTCCA  Reverse: ATACTGGTCTGTTGTGGGTGG |
| NF-κβ | Forward: CATGAAGAGAAGACACTGACCATGGAA  Reverse: TGGATAGAGGCTAAGTGTAGACACG |
| TNF-α | Forward: TCCACGCTCCTTCTGTCACTG  Reverse: CTTGGTGGTTTGCTACGAC |
| GAPDH | Forward: GGTGGACCTCATGGCCTACAT  Reverse: GCCTCTCTCTTGCTCTCAGTATCCT |
